# In Vitro Characterization of Vaccine Strain-like Porcine Reproductive and Respiratory Syndrome Virus Strains Isolated from Weaned Pigs Exhibiting Respiratory Symptoms

**DOI:** 10.3390/pathogens14100990

**Published:** 2025-10-01

**Authors:** Inori Goda, Akiha Inoue, Isshu Kojima, Mana Esaki, Taichi Hasegawa, Kosuke Okuya, Makoto Ozawa

**Affiliations:** 1 Joint Faculty of Veterinary Medicine, Kagoshima University, Kagoshima 890-0065, Japan; d241005@edu.kaiyodai.ac.jp (I.G.); k0307138@kadai.jp (A.I.); kokuya@vet.kagoshima-u.ac.jp (K.O.); 2 Joint Graduate School of Veterinary Medicine, Kagoshima University, Kagoshima 890-0065, Japan; i-kojima@ous.ac.jp (I.K.); k0068103@kadai.jp (M.E.); 3 Matsuoka Research Institute for Science, Koganei 184-0003, Japan; taichi.hasegawa@matsuken-yakuhin.com

**Keywords:** porcine reproductive and respiratory syndrome virus, vaccine strain, point mutation

## Abstract

Porcine reproductive and respiratory syndrome virus (PRRSV) infection causes significant economic losses in swine production. In May 2021 and March 2023, we detected PRRSV genes in serum samples from two weaned pigs with respiratory disorders on a farm in Japan. Partial gene sequences of these strains closely resembled those of a PRRS vaccine strain. We subsequently isolated two PRRSV field strains, KU-IG21-1 and KU-IG23-1, from the 2021 and 2023 samples, respectively. The KU-IG21-1 strain exhibited more pronounced cytopathic effects and significantly higher replication efficiency in cultured cells compared to both the vaccine and KU-IG23-1 strains. Despite these phenotypic differences, complete genome sequencing revealed high genetic similarity between the field isolates and the vaccine strain, with only 16 and 24 amino acid differences in the KU-IG21-1 and KU-IG23-1 strains, respectively. These findings suggest that the field strains likely emerged through the accumulation of point mutations in the vaccine strain rather than through homologous recombination. Furthermore, we identified three amino acid substitutions that may contribute to the enhanced replication of the KU-IG21-1 strain. This study underscores the potential impact of point mutations on PRRSV phenotypes and provides new insights into the complex evolutionary dynamics of PRRSV.

## 1. Introduction

Porcine reproductive and respiratory syndrome (PRRS) is a viral disease caused by the PRRS virus (PRRSV) that primarily leads to reproductive failure in sows and respiratory distress in both growing and adult pigs [1]. PRRSV infection induces prolonged viremia lasting over two months, complicating efforts to eradicate the virus from pig farms [2,3]. Moreover, PRRSV-induced immunosuppression increases the susceptibility to secondary infections, often resulting in increased mortality among infected pigs [4,5]. As a significant cause of economic loss in the swine industry, PRRS has been estimated to incur annual costs ranging from € 75,724 to € 650,090 [6]. Despite extensive efforts, there are no effective treatments for PRRSV-infected pigs, making preventive measures such as vaccination essential. Several live-attenuated and killed PRRS vaccines are currently available in various countries, including Japan.

PRRSV was identified as the causative agent of PRRS nearly simultaneously in North America and Europe in the late 1980s [7]. The European and North American PRRSV isolates were soon recognized as genetically distant [8,9,10] and were subsequently classified into two genotypes: genotype 1 (PRRSV-1) and genotype 2 (PRRSV-2) [10]. PRRSV belongs to the family Arteriviridae in the order Nidovirales and is a positive-stranded RNA virus with a genome of approximately 15 kb [11]. Its genome structure includes a 5′ untranslated region (5′-UTR), non-structural protein-coding genes (ORF1a and ORF1b genes), structural protein-coding genes (ORF2 to ORF7 genes), a 3′-UTR, and a poly (A) tail [12]. Among these, the ORF5 gene, which encodes glycoprotein 5 (GP5), a major viral envelope protein [13], is highly prone to genetic variations, making it an important target for phylogenetic analysis [14,15]. Conversely, the ORF6 and ORF7 genes, which encode the matrix protein (M) and nucleoprotein (N), respectively [13], are used as targets in polymerase chain reaction (PCR) diagnostics because of their relative genetic stability and lower mutation rates [16,17].

Similar to other RNA viruses, PRRSV is prone to frequent mutations [18]. PRRSV mutants can emerge not only through point mutations but also via homologous recombination between different viral strains [19,20]. Various homologous recombinants, including those produced between vaccines and field strains, have been reported worldwide [21,22]. Consequently, the use of live-attenuated PRRSV vaccines raises concerns regarding the potential generation of revertants or novel, more pathogenic mutants.

In pigs, PRRSV is typically detected through viral gene-specific quantitative reverse transcription polymerase chain reaction (qRT-PCR) performed on serum, lung tissue, or oral fluid samples. Field strains often exhibit more vigorous replication in pigs than vaccine strains, leading to a higher number of viral gene copies in these samples. Positive samples are then subjected to nucleotide sequencing, usually targeting the ORF5 gene, to distinguish between the vaccine and field strains. However, determining the virus strain based solely on the ORF5 gene is challenging because of its potential for homologous gene recombination [23,24]. Whole-genome sequencing is necessary to comprehensively understand the genetic characteristics of this virus [25,26].

In May 2021 and March 2023, serum samples were collected from two 12-week-old weaned pigs with respiratory disorders characterized by emaciation and rough coats at a farm in Kagoshima Prefecture, Japan. Although the pigs received the PRRS vaccine at 14 days of age, qRT-PCR analysis detected PRRSV genes in the serum, suggesting a possible infection with field strains. However, sequence analysis of the ORF5 genes revealed that the PRRSVs detected in both samples were nearly identical to one of the commercial PRRS vaccine strains used on the farm. These findings suggest that the detected PRRSVs may be unique genetic variants of the vaccine strain, potentially resulting from homologous recombination with field strains. In this study, we isolated these two vaccine-like strains and examined their replicative capabilities in cultured cells as well as their genetic characteristics through whole-genome sequencing.

## 2. Materials and Methods

### 2.1. Cells and Viruses

African green monkey kidney-derived MA-104 cells (kindly provided by Vaxxinova Japan K.K., Tokyo, Japan) were cultured in Eagle’s minimal essential medium (E-MEM; FUJIFILM Wako Pure Chemical Corporation, Osaka, Japan) supplemented with 10% fetal bovine serum (FBS), 100 U/mL penicillin, 100 μg/mL streptomycin, and 0.25 μg/mL amphotericin B, maintained at 37 °C in 5% CO_2_.

Porcine alveolar macrophage-derived PAM-T43 cells, established in our laboratory [27], were cultured in Roswell Park Memorial Institute 1640 medium (FUJIFILM Wako Pure Chemical Corporation) supplemented with 10% FBS, 10% porcine serum, 100 U/mL penicillin, 100 μg/mL streptomycin, 0.25 μg/mL amphotericin B, 1% MEM nonessential amino acids solution (Nacalai Tesque, Inc., Kyoto, Japan), 1% L-sodium pyruvate solution (Nacalai Tesque, Inc.), and 1% L-HEPES buffer solution (Nacalai Tesque, Inc.), maintained at 37 °C in 5% CO_2_.

The PRRS vaccine strain, Ingelvac PRRS MLV (Boehringer Ingelheim Animal Health Inc., Ingelheim am Rhein, Germany), administered to weaned pigs, was propagated in MA-104 cells and stored at −80 °C until use. Two PRRSV field strains were isolated during this study (details provided below).

### 2.2. Samples

Serum samples were collected from two weaned pigs exhibiting respiratory disorders on a commercial farm in Kagoshima Prefecture, Japan, in May 2021 and March 2023. The samples were obtained primarily for diagnostic purposes and subsequently subjected to PRRSV isolation. Quantitative reverse transcription PCR (qRT-PCR), as previously described [28], was applied to confirm the presence of PRRSV genes in the samples.

### 2.3. Virus Isolation

Following filter sterilization with 0.22 μm pore filters (Sartorius AG, Göttingen, Germany), the serum samples were inoculated into PAM-T43 cells and cultured for 3–4 days. PRRSV isolation was confirmed by observing virus-induced cytopathic effects (CPEs) in cultured cells under an Eclipse Ti-S fluorescent microscope (Nikon, Tokyo, Japan) and subsequent qRT-PCR detection of high viral loads (Ct values < 20) of PRRSV genes in the culture supernatants. The isolated PRRSV field strains were then propagated in PAM-T43 cells and stored at −80 °C until further use.

### 2.4. Plaque Formation Assays with Immunostaining

MA-104 and PAM-T43 cells were inoculated with each PRRSV strain and overlaid with growth medium containing 0.6% Avicel. At 2 days post-infection (dpi), the cells were fixed with Mildform (FUJIFILM Wako Pure Chemical Corporation) and subjected to immunostaining using a monoclonal antibody specific to the PRRSV N protein (clone SR30-A; RTI LLC, Brookings, SD, USA). A DyLight 488-conjugated anti-mouse IgG (H + L) secondary antibody (Vector Laboratories, Newark, CA, USA) was then applied. Resulting plaques were visualized under the Eclipse Ti-S fluorescent microscope.

### 2.5. Virus Titration

Viral titers were determined using 50% tissue culture infective dose (TCID_50_) assays in MA-104 and PAM-T43 cells. Briefly, MA-104 cells (2500 cells/well) or PAM-T43 cells (25,000 cells/well) were seeded in 96-well plates and, after 24 h, inoculated with ten-fold serial dilutions of the virus, with four wells per dilution. The inoculated cells were incubated at 37 °C for 4–6 days, after which they were stained with crystal violet (Supplementary Figure S1). Wells that remained unstained with crystal violet were considered infected with the virus. The TCID_50_ titers were calculated using the Reed and Muench method [29].

### 2.6. Virus Replication Kinetics

MA-104 or PAM-T43 cells at 80% confluence were seeded in 6-well plates overnight, and each viral strain was inoculated at a multiplicity of infection (MOI) of 0.01. After viral infection, 20 µL of cell culture supernatants was collected at 24 h intervals for virus titration. TCID_50_ titers were calculated using the Reed and Muench method as described above.

### 2.7. RNA Extraction

Culture supernatants containing propagated PRRSVs were clarified through centrifugation at 4000 rpm for 15 min at 4 °C using an Allegre X-30R Centrifuge (Beckman Coulter, Inc., Pasadena, CA, USA). The clarified supernatants were then filtered through 0.22-μm pore filters (Sartorius AG). To minimize contamination with nucleic acids derived from host cells or other organisms—which could interfere with accurate viral genome determination by next-generation sequencing—viruses were concentrated through ultracentrifugation over a 15% sucrose cushion (40,000 rpm, 2 h, 4 °C) using a CS-120GX (Hitachi High-Technologies Corporation, Tokyo, Japan) and subsequently resuspended in phosphate-buffered saline. RNA was extracted from the purified PRRSVs using the innuPREP Virus DNA/RNA kit (IST Innuscreen GmbH, Berlin, Germany) according to the manufacturer’s instructions, and the extracted RNA was stored at −80 °C until future use.

### 2.8. Nanopore Sequencing

The PrimeScript Double Strand cDNA Synthesis Kit (Takara Bio Inc., Kusatsu, Japan) was used to synthesize double-stranded cDNA from RNA extracted from the purified PRRSVs. The synthesized double-stranded cDNA was purified using the innuPREP PCR pure Lite kit (IST Innuscreen GmbH) and stored at −20 °C until future use.

Double-stranded cDNAs were barcoded using PCR Barcoding Expansion 1–12 (Oxford Nanopore Technologies, Oxford, UK) and loaded onto a MinION Flow Cell (R10 version; Oxford Nanopore Technologies) for nanopore sequencing according to the manufacturer’s instructions. Nucleotide sequences obtained from the field strains were compared with those of the MLV vaccine strain (GenBank Accession No. AF066183.4) using Geneious Prime version 2023.02 (Dotmatics, Woburn, MA, USA).

### 2.9. Determination of the 5′ Terminal Sequence of PRRSV Genome

The 5′ terminal sequences of PRRSV genome were determined by 5′ rapid amplification of cDNA ends (5′ RACE). cDNA was synthesized from RNA extracted from the purified PRRSVs using SuperScript IV Reverse Transcriptase (Thermo Fisher Scientific, Waltham, MA, USA) and the virus-specific primer PRRSV-846R (Table 1), targeting a sequence approximately 850 nucleotides downstream from the 5′ end of the viral genome. This step was followed by treatment with RNaseH (Nippon Gene Co., Ltd., Tokyo, Japan) for 20 min at 37 °C. The cDNA was purified using the innuPREP PCR pure Lite kit (IST Innuscreen GmbH) and then ligated with a 3′ end cordycepin-blocked adaptor DT88 (Table 1) [30] using T4 RNA ligase (New England Biolabs, Ipswich, MA, USA). PCR amplification was subsequently performed using KOD One PCR Master Mix -Blue- (Toyobo Co., Ltd., Osaka, Japan) with the second virus-specific primer PRRSV-687R (Table 1), targeting a sequence of approximately 690 nucleotides downstream from the 5′ end of the viral genome, and the complementary primer DT89 (Table 1) corresponding to the adaptor DT88.

The PCR products were separated on a 1% agarose gel using electrophoresis, purified using the Wizard SV Gel and PCR Clean-up System (Promega, Madison, WI, USA), and subsequently cloned into the pCR Blunt II-TOPO plasmid vector using the Zero Blunt TOPO PCR Cloning Kit (Thermo Fisher Scientific). Four plasmid clones for each viral strain were randomly selected, and the nucleotide sequences of the cloned PCR products were determined through Sanger sequencing at Azenta Japan Corp. (Tokyo, Japan). The sequences obtained were analyzed using MEGA software version X [31].

### 2.10. Determination of the 3′ Terminal Sequence of PRRSV Genome

The 3′ terminal sequences of PRRSV genome were determined by 3′ RACE. cDNA was synthesized from RNA extracted from the purified PRRSVs using SuperScript IV Reverse Transcriptase (Thermo Fisher Scientific) and the primer DT88 + T24, which includes the DT88 adaptor sequence followed by 24 consecutive thymine bases. The adaptor-ligated cDNA was then amplified using PCR with a second virus-specific primer, PRRSV-M-F (Table 1), which corresponds to a sequence approximately 740 nucleotides upstream from the 3′ end of the viral genome, along with the adaptor DT88. The resulting PCR products were cloned into a plasmid vector and subjected to Sanger sequencing, as described above, for the 5′ RACE procedure.

## 3. Results

### 3.1. Cytopathic Effect by the Field Isolates

**Figure 1:**
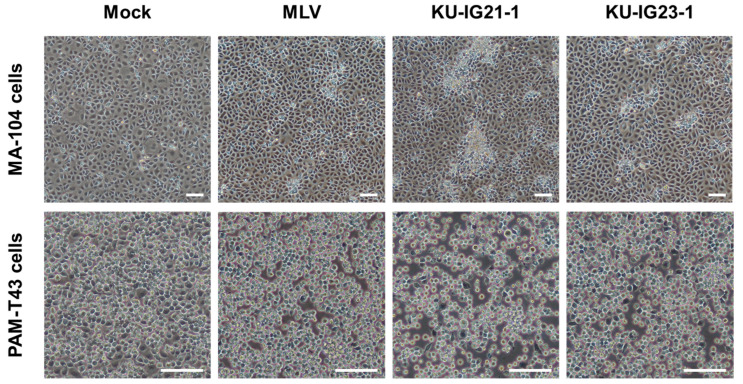
Representative microscopic images of cultured cells infected with three PRRSV strains. MA-104 and PAM-T43 cells were inoculated with the vaccine strain MLV or the field strains KU-IG21-1 and KU-IG23-1 at an MOI of 0.1. Infected cells were observed under a microscope at 42 dpi. Scale bars, 200 μm.

The two PRRSV isolates derived from weaned pigs with respiratory disorders on a farm in Kagoshima Prefecture, Japan, in May 2021 and March 2023 were designated as KU-IG21-1 and KU-IG23-1 strains, respectively (Supplementary Figure S2). During propagation, the KU-IG21-1 strain exhibited a more pronounced CPE in MA-104 cells than the vaccine strain MLV and another field isolate, KU-IG23-1 (Figure 1). Although differences in CPE among the viral strains in PAM-T43 cells were not as evident (Figure 1), these observations suggest that the KU-IG21-1 strain may possess distinct in vitro characteristics compared with other PRRSV strains.

### 3.2. Replication Kinetics of the Field Isolates

**Figure 2:**
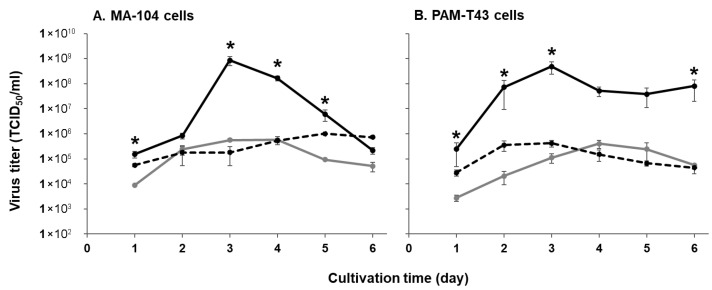
Replication kinetics of three PRRSV strains in cultured cells. MA-104 (**A**) and PAM-T43 (**B**) cells were infected with the vaccine strain MLV (gray lines) or the field strains KU-IG21-1 (black lines) and KU-IG23-1 (dashed black lines) at an MOI of 0.1. Viral titers in the culture supernatants collected on the indicated days were determined using TCID_50_ assays in the corresponding cell lines. Error bars represent the standard error of triplicate experiments. Comparisons between the vaccine strain and each field strain at each time point were conducted, and *p*-values were calculated using the Mann–Whitney U test. *p*-values less than 0.05 were considered statistically significant and are indicated by an asterisk (*).

To evaluate the replicative capability of the field isolates KU-IG21-1 and KU-IG23-1 compared with the PRRS vaccine strain MLV, we examined their growth kinetics in two representative cell lines for PRRS research: MA-104 and PAM-T43 cells. Viral presence in the culture supernatants was detected as early as 1 dpi, with all strains reaching peak titers at 3 dpi. In both MA-104 and PAM-T43 cells, the viral titers of the KU-IG21-1 field strain were significantly higher than those of the vaccine strain at most time points, and the viral titer of the KU-IG21-1 strain in MA-104 cells at 5 dpi was over 1,000 times higher than that of the vaccine strain (Figure 2). In contrast, the growth kinetics of the KU-IG23-1 field strain were comparable to those of the vaccine strain (Figure 2). These results indicate that the replicative capability of the KU-IG21-1 strain was markedly superior to that of the vaccine and KU-IG23-1 strains.

### 3.3. Genome Analysis of the Field Isolates

The nucleotide sequences of the ORF5 gene from the field isolates KU-IG21-1 and KU-IG23-1 were nearly identical to those of the vaccine strain. However, the KU-IG21-1 strain exhibited a significantly higher replicative capability than the vaccine strain in both tested cell lines, suggesting the potential presence of critical mutations, possibly including homologous gene recombination events, between the vaccine and field strains. To investigate the genetic characteristics of the vaccine strain and the two field strains, their complete genome sequences were determined. While nearly the entire genome sequences were determined through nanopore sequencing, the terminal sequences at both ends of the genome were determined using RACE and subsequently confirmed by Sanger sequencing. The combined sequencing results revealed that the complete genome sequences of all tested viral strains were nearly identical (Table 2). These findings indicate that neither the KU-IG21-1 strain nor the KU-IG23-1 strain is a product of homologous gene recombination between the vaccine and field strains. The full-length genome sequences of the KU-IG21-1 and KU-IG23-1 strains have been deposited in the GenBank database under Accession Nos. PX097596 and PX097597, respectively.

To identify the amino acids that potentially affect the replicative capability of PRRSVs, we compared the deduced amino acid sequences of the vaccine strain and the two field strains. The comparison revealed scattered amino acid differences throughout the coding sequence of the field strains relative to that of the vaccine strain, providing no evidence of homologous gene recombination events in the genome of the field strains. Specifically, the KU-IG21-1 strain exhibited 16 amino acid substitutions compared with the vaccine strain (Table 3), suggesting that these substitutions could potentially contribute to its superior replicative capability. Conversely, the KU-IG23-1 strain, which exhibited a replicative capacity similar to that of the vaccine strain, contained 24 amino acid substitutions (Table 3), indicating that these substitutions had a limited impact on its replicative capability.

## 4. Discussion

In this study, we isolated two PRRSV field strains, KU-IG21-1 and KU-IG23-1, from weaned pigs with respiratory disorders. The KU-IG21-1 strain demonstrated a more pronounced CPE (Figure 1) and a significantly higher replicative capability in cultured cells than the vaccine and KU-IG23-1 strains (Figure 2). Despite these phenotypic differences, the complete genome sequences showed that the two field isolates and vaccine strain exhibited a high degree of genetic similarity (Table 2). Comparative analysis of the deduced amino acid sequences revealed that the KU-IG21-1 and KU-IG23-1 strains differed from the vaccine strain by 16 and 24 amino acid substitutions, respectively (Table 3).

Among the 16 amino acid substitutions identified in the KU-IG21-1 strain, which exhibited superior replicative capability compared with the vaccine strain, seven substitutions were also found in the KU-IG23-1 strain, which displayed replicative capability similar to that of the vaccine strain (Table 3). Therefore, the remaining nine substitutions, i.e., glutamine to arginine substitution at position 103 of Nsp1β protein (Nsp1β-Q103R), Nsp2-R27W, Nsp2-T353I, Nsp2-N723D, Nsp2-I985V, GP2-N188S, GP2-L252P, GP5-N58D, and M-H10R, are likely contributors to the enhanced replicative capability of the KU-IG21-1 strain.

The Nsp1β protein, a non-structural protein of PRRSV, functions as a protease [32] and is known for its high genetic conservation [33]. This viral protein interacts with the host immune system and is linked to the intracellular stress granules [34,35]. Therefore, the substitution Nsp1β-Q103R may play a critical role in the enhanced replicative capability observed in the KU-IG21-1 strain.

The Nsp2 protein, a viral membrane protein, shares its N-terminal region, including amino acid positions 27 and 353, with both Nsp2TF and Nsp2N proteins [36,37]. These two accessory proteins are expressed in the same open reading frame as the Nsp2 protein through ribosomal frameshifting in downstream regions. The N-terminal region common to the Nsp2, Nsp2TF, and Nsp2N proteins plays a role in modulating innate immunity [34,38]. Consequently, two of the four amino acid substitutions detected in Nsp2 in this study, Nsp2-R27W and Nsp2-T353I, may have contributed to the enhanced replicative capability of the KU-IG21-1 strain. The third and fourth substitutions, Nsp2-I985V and Nsp2-N723D, are located in the central region of the Nsp2 protein, which is characterized by frequent large variations such as deletions [39,40] and are prone to mutations [41].

The GP2 protein is one of the minor structural proteins in the viral envelope that forms a complex with GP3 and GP4 proteins [12]. Because of the presence of functional domains essential for complex formation throughout its structure, the GP2 protein is generally considered genetically stable [42]. Although there is limited information on its intracellular functions, a previous study has suggested that the GP2 protein might play a role in inhibiting PRRSV-induced apoptosis [43]. Although specific amino acids within the GP2 protein have been linked to cell tropism [44], the two amino acid substitutions identified in this study, GP2-N188S and GP2-L252P, were not previously associated with this function.

The GP5 protein, which forms a complex with the M protein and serves as a major structural component of the viral envelope [12], plays a crucial role in viral survival [45]. Among all PRRSV proteins, the GP5 protein is the most susceptible to variation [46,47] and contains numerous antigenic determinants [48]. Therefore, the amino acid substitution identified in this study, GP5-N58D, is unlikely to contribute significantly to the superior replicative capability of the KU-IG21-1 strain despite not being previously identified in variants analyzed in earlier studies [48,49].

The M protein, a non-glycosylated structural protein, exhibits the highest degree of genetic conservation among the structural proteins of arteriviruses, including PRRSV [50,51]. However, the specific substitution M-H10R found in the KU-IG21-1 strain occurred in a region characterized by relatively high variability. This site is one of two B-cell epitopes within the M protein [52].

Overall, of the nine amino acid substitutions unique to the KU-IG21-1 strain, Nsp1β-Q103R, Nsp2-R27W, and/or Nsp2-T353I are more likely to be associated with its superior replicative capability. To validate this hypothesis, further molecular virological investigations, such as those employing reverse genetics, are necessary.

Although the KU-IG21-1 strain demonstrates superior replicative capability in cultured cells, its association with clinical symptoms remains unclear. Several in vitro studies have sought to elucidate the pathogenicity of individual PRRSV strains; however, it is unlikely that pathogenicity can be reliably assessed solely using in vitro assays [53,54]. Although the KU-IG21-1 strain was isolated from weaned pigs exhibiting respiratory disorders, it is possible that the observed clinical symptoms were due to coinfection with other pathogens [55,56]. As this study did not include comprehensive testing, the influence of coinfections cannot be excluded. Furthermore, the virus isolations from 12-week-old pigs vaccinated at 14 days of age represent unusual findings, as the vaccine strain analyzed in this study is typically detectable in serum for up to six weeks post-vaccination [57]. Consequently, to confirm the pathogenicity of the KU-IG21-1 strain, further studies involving in vivo experiments are necessary.

These findings also provide important insights for PRRS control programs. The isolation of a vaccine-derived strain with enhanced in vitro replicative capability underscores the need for careful interpretation of diagnostic results, particularly in vaccinated herds. Although the detection of vaccine-like strains in older pigs is generally considered unusual, our study highlights the critical importance of continuous genomic monitoring to distinguish vaccine-derived viruses from potentially emerging field variants. From a practical perspective, these results emphasize the necessity of integrating molecular surveillance with vaccination programs to facilitate the early detection of atypical viral variants and to guide timely adjustments in vaccination and biosecurity strategies.

## 5. Conclusions

One of the two PRRSV field strains isolated from weaned pigs with respiratory disorders exhibited superior replicative capability in cultured cells. Genome analysis indicated that the field strain emerged through the accumulation of point mutations within the vaccine strain rather than via homologous gene recombination between different virus strains. A comparative analysis of the deduced amino acid sequences identified three specific substitutions that may have contributed to their enhanced replicative capability. These findings highlight the potential impact of point mutations on PRRSV characteristics and offer valuable insights into the complex evolutionary dynamics of PRRSV.

## Figures and Tables

**Table 1:** Primers used in this study.

Name	Nucleotide Sequence (5′ to 3′)	Position *	Reference
PRRSV-846R	GAGCCGGTTCGCAATCAACT	865 to 846	This study
PRRSV-687R	GTCTTCAGGCTTGGGTCTCT	706 to 687	This study
PRRSV-M-F	TTGTGCTTGCTAGGCCGCA	14,676 to 14,694	This study
DT88	GAAGAGAAGGTGGAAATGGCGTTTTGG	(adaptor oligonucleotide)	[30]
DT89	CCAAAACGCCATTTCCACCTTCTCTTC	(adaptor oligonucleotide)	[30]

* Position of primers is given according to the nucleotide sequence numbering of MLV vaccine strain in the GenBank database (AF066183.4).

**Table 2:** Nucleotide and amino acid sequence similarity between the vaccine strain and the two field strains isolated in this study.

Gene	Sequence Similarity to the Counterpart from the Vaccine Strain of:			
	KU-IG21-1 Strain in:		KU-IG23-1 Strain in:	
	Nucleotide	Amino Acid	Nucleotide	Amino Acid
5′-UTR	98.95	- *	100.0	-
ORF1a gene	99.79	99.76	99.73	99.56
ORF1b gene	99.91	99.93	99.89	99.86
ORF2 gene	99.61	99.22	99.48	98.83
ORF3 gene	99.48	98.82	99.22	98.04
ORF4 gene	99.63	100.0	99.63	100.0
ORF5 gene	99.34	99.00	99.34	99.00
ORF6 gene	99.62	98.86	99.81	99.43
ORF7 gene	99.73	100.0	100.0	100.0
3′-UTR	100.0	-	99.34	-
Entire gene	99.70%	99.77%	99.66%	99.61%

* not applicable.

**Table 3:** Amino acid differences between the PRRS vaccine strain and the two field strains isolated in this study.

Protein	Position	Amino Acid From:		
		Vaccine Strain	KU-IG21-1 Strain	KU-IG23-1 Strain
Nsp1β	103	Q	R	-
	141	L	- *	P
	151	F	S	S
	169	S	-	P
Nsp2	27	R	W	-
	63	A	-	V
	353	T	I	-
	361	K	-	T
	558	S	-	N
	632	I	-	F
	723	N	D	D
	827	G	-	(deletion)
	985	I	V	-
	1122	K	-	E
Nsp4	279	I	-	M
Nsp9	108	H	-	Y
Nsp10	306	H	Y	Y
GP2	10	F	-	L
	122	A	-	S
	188	N	S	-
	237	I	-	M
	252	L	P	-
GP3	30	T	-	A
	63	T	A	-
	85	D	-	N
	94	I	V	V
	176	G	-	D
	215	I	T	T
GP5	10	C	-	Y
	58	N	D	-
	151	G	I	I
M	10	H	R	-
	16	E	Q	Q

* Amino acid identical to its counterpart in the vaccine strain.

## Data Availability

The original contributions presented in this study are included in the article and Supplementary Materials. Further inquiries can be directed to the corresponding author(s).

## Supplementary Materials

The following supporting information can be downloaded at: https://www.mdpi.com/article/10.3390/pathogens14100990/s1, Figure S1: Representative images of crystal violet-stained MA-104 and PAM-T43 cells used for virus titration.; Figure S2: Representative plaques formed by three PRRSV strains and visualized by immunostaining with a PRRSV-specific monoclonal antibody.
