# Supplementary material for: In Vitro Characterization of Vaccine Strain-like Porcine Reproductive and Respiratory Syndrome Virus Strains Isolated from Weaned Pigs Exhibiting Respiratory Symptoms
Source: Pathogens. 2025 Oct 1;14(10):990. doi: 10.3390/pathogens14100990 (PMC12567286; doi:10.3390/pathogens14100990)
Supplement: Supplementary file 1 [file pathogens-14-00990-s001.zip › pathogens-3836385-supplementary.pdf]

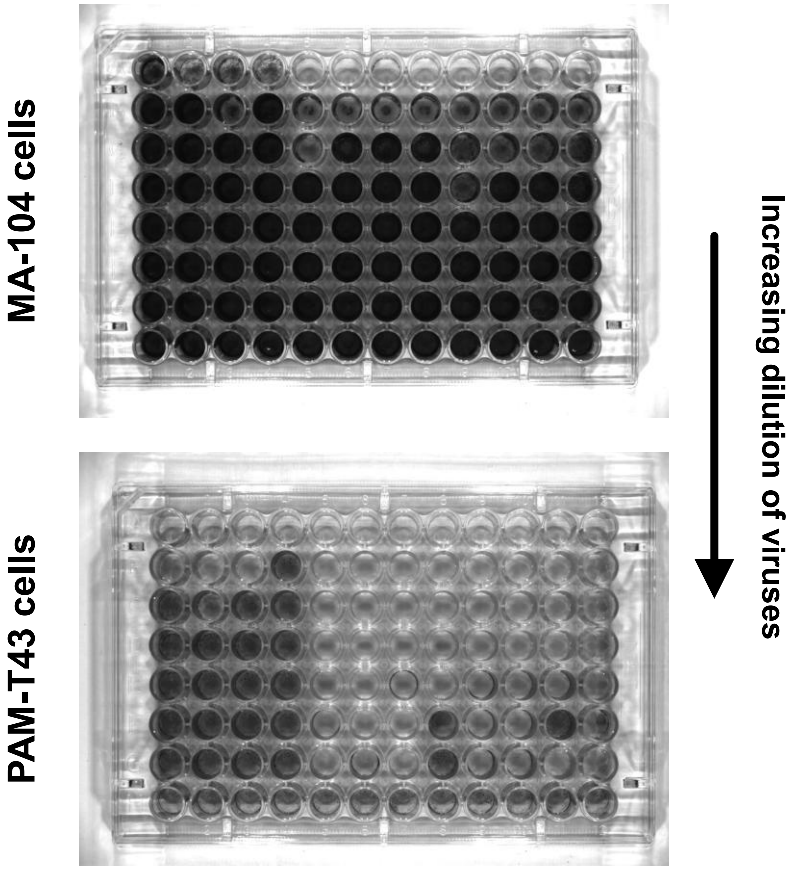

Supplementary Figure S1. Representative images of crystal violet-stained MA-104 and PAM-T43 cells used for virus titration. Cells were inoculated with ten-fold serial dilutions of each PRRSV strain, with four replicate wells per dilution. After incubation (6 days for MA-104 cells and 4 days for PAM-T43 cells), cells were fixed and stained with crystal violet to visualize cytopathic effects.

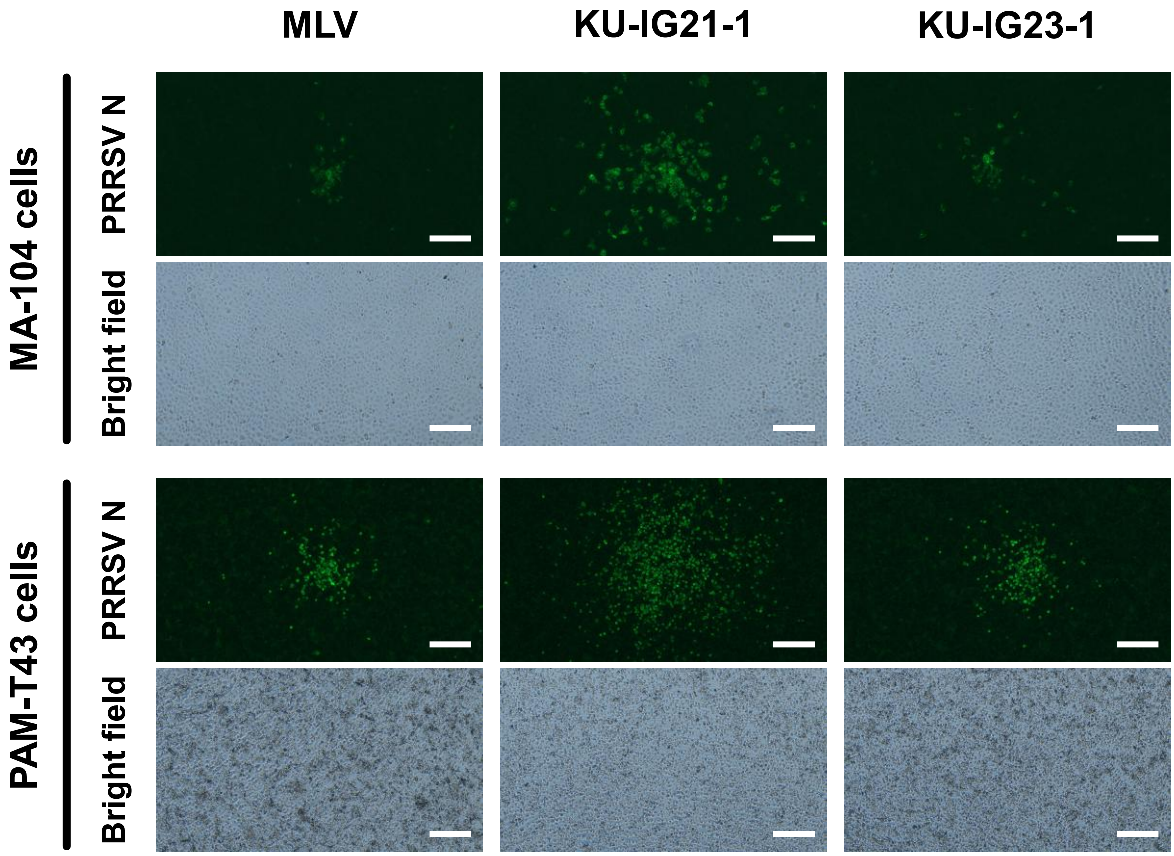

Supplementary Figure S2. Representative plaques formed by three PRRSV strains and visualized by immunostaining with a PRRSV-specific monoclonal antibody. Scale bars, 400 μm.
